# Supplementary material for: Content-rich biological network constructed by mining PubMed abstracts
Source: BMC Bioinformatics. 2004 Oct 8;5:147. doi: 10.1186/1471-2105-5-147 (PMC528731; doi:10.1186/1471-2105-5-147)
Supplement: Additional File 2 — The original results of the above study (non-essential files are deleted to keep the file size under the limit set by BMC bioinformatics). [file 1471-2105-5-147-S2.bz2 › chilibotAdditionalFile2/dip10/559ID10395669E10919/html/IFNAR2_IFNB1.html]

 


 **IFNAR2** and **IFNB1** 
  
Found 32 abstracts in PubMed, retrieved 10.  
 

 What does Google say? 
 PDF only 
| .edu only 

---

**Interactive relationship** (e.g. stimulation, inhibition, etc)

**Inhibitory relationship**- However, two mAb that bound to sites remote from the IFNAR 2  [ **IFNAR2** ]  binding site on IFN beta  [ **IFNB1** ]  also inhibited IFN beta  [ **IFNB1** ]  IFNAR 2  [ **IFNAR2** ]  complex formation and demonstrated potent neutralizing activity.  Ref: 11747625 J Interferon Cytokine Res, 2001
**Neutral relationship**- The human type I interferons, IFN alpha, IFN beta  [ **IFNB1** ] , and IFN omega, induce somewhat different cellular effects but act through a common receptor complex, IFNAR, composed of subunits IFNAR 1 and IFNAR 2  [ **IFNAR2** ] .  Ref: 11278538 J Biol Chem, 2001

**Non-interactive relationship** (e.g. studied together, co-existance, homology, etc.)

- Two of these inhibited IFN beta  [ **IFNB1** ]  IFNAR 2  [ **IFNAR2** ]  complex formation, suggesting that steric hindrance of receptor binding constitutes their mechanism of neutralization.  Ref: 11747625 J Interferon Cytokine Res, 2001
- A panel of monoclonal antibodies mAb derived against human interferon alpha beta receptor 2  [ **IFNAR2** ]  IFNAR 2  [ **IFNAR2** ]  was evaluated for their ability to antagonize the biologic effects of type 1 interferons IFN alpha1, IFN alpha2a, and IFN beta  [ **IFNB1** ] .  Ref: 11096454 J Interferon Cytokine Res, 2000
